# A 20-Amino-Acid Deletion in the Neuraminidase Stalk and a Five-Amino-Acid Deletion in the NS1 Protein Both Contribute to the Pathogenicity of H5N1 Avian Influenza Viruses in Mallard Ducks

**DOI:** 10.1371/journal.pone.0095539

**Published:** 2014-04-17

**Authors:** Yanfang Li, Sujuan Chen, Xiaojian Zhang, Qiang Fu, Zhiye Zhang, Shaohua Shi, Yinbiao Zhu, Min Gu, Daxin Peng, Xiufan Liu

**Affiliations:** College of Veterinary Medicine, Yangzhou University, Jiangsu Co-Innovation Center for the Prevention and Control of Important Animal Infectious Disease and Zoonoses, Yangzhou University, Yangzhou, Jiangsu, P.R. China; The University of Hong Kong, Hong Kong

## Abstract

Since 2003, H5N1-subtype avian influenza viruses (AIVs) with both a deletion of 20 amino acids in the stalk of the neuraminidase (NA) glycoprotein (A−) and a deletion of five amino acids at positions 80 to 84 in the non-structural protein NS1 (S−) have become predominant. To understand the influence of these double deletions in the NA and NS1 proteins on the pathogenicity of H5N1-subtype AIVs, we selected A/mallard/Huadong/S/2005 as a parental strain to generate rescued wild-type A−S− and three variants (A−S+ with a five-amino-acid insertion in the NS1 protein, A+S− with a 20-amino-acid insertion in the NA stalk, and A+S+ with insertions in both NA and NS1 proteins) and evaluated their biological characteristics and virulence. The titers of the AIVs with A− and/or S− replicated in DEF cells were higher than that of A+S+, and the A−S− virus exhibited a replication predominance when co-infected with the other variants in DEF cells. In addition, A−S− induced a more significant increase in the expression of immune-related genes in peripheral blood mononuclear cells of mallard ducks *in vitro* compared with the other variants. Furthermore, an insertion in the NA and/or NS1 proteins of AIVs resulted in a notable decrease in virulence in ducks, as determined by intravenous pathogenicity index, and the two insertions exerted a synergistic effect on the attenuation of pathogenicity in ducks. In addition, compared with A+S+ and A+S−, the A−S+ and A−S− viruses that were introduced via the intranasal inoculation route exhibited a faster replication ability in the lungs of ducks. These data indicate that both the deletions in the NA stalk and the NS1 protein contribute to the high pathogenicity of H5N1 AIVs in ducks.

## Introduction

Avian influenza virus has a wide geographical distribution in poultry and wild birds and certain genotypes/subtypes exhibit continuous cross-species transmission to humans and other mammals, which has resulted in the global concern of a potential pandemic threat [1]. The viral surface glycoproteins hemagglutinin (HA) and neuraminidase (NA) are major determinants in the interspecies transmission and adaptation of influenza A viruses to a new host [2]. The sialidase activity of NA not only facilitates the release and diffusion of progeny virions but also initiates the viral infection process [3]–[5]. A deletion in the stalk region of the NA (A−) decreases the ability of NA to release the virus from cells [6]–[9] and alters the virulence of the virus [10], [11]. In addition, a deletion in the stalk of the NA gene may be required for the adaptation of H5N1 influenza viruses from wild aquatic birds to poultry [12]–[19].

The non-structural (NS) gene of influenza A virus encodes two proteins, namely NS1 and NEP, which share ten amino acids from the first residues at the N-terminal of the ORF [20]. The NS1 protein is a multifunctional protein involved in various protein-protein and protein-RNA interactions. In addition, NS1 is responsible for the inhibition of host immune responses by regulating the production of interferons (IFN) in the infected cells [21]–[23], the downregulation of host apoptosis, the post-transcriptional block of cellular mRNA maturation [24], and the regulation of the pathogenicity of influenza A viruses [25], [26]. A five-amino-acid deletion at positions 80 to 84 in the NS1 protein of H5N1-subtype AIVs (S−) appeared in 2000 [16], [27]–[29], which has resulted in an increase in the virulence of H5N1 viruses in chicken and mice [30].

H5N1 influenza viruses with both a short NA stalk and a five-amino-acid deletion in the NS1 protein were first found in 2002 and were the prevailing strains by 2003. However, the role of the double deletions in the NA and NS1 proteins in the pathogenicity of H5N1 subtype AIVs remains unknown. In this study, four rescue viruses with or without deletions in the NA and NS1 proteins were obtained using a reverse genetics technique based on the wild-type H5N1-subtype AIV strain A/mallard/Huadong/S/2005, and their biological characteristics and virulence were determined.

## Materials and Methods

### Ethics Statement

All of the animal studies were approved by the Jiangsu Administrative Committee for Laboratory Animals (Permission number: SYXKSU-2007-0005) and complied with the guidelines for laboratory animal welfare and ethics of the Jiangsu Administrative Committee for Laboratory Animals.

### Viruses and Cells

A/mallard/Huadong/S/2005(SY), which has a 20-amino-acid deletion in the NA stalk and a five-amino-acid deletion at residues 80–84 in the NS1 protein, was isolated from mallard ducks and identified as an H5N1-subtype highly pathogenic AIV by our lab [31]. MDCK, 293T, and Vero cells were purchased from the Shanghai Institute of Biological Science, CAS, and cultured in DMEM (Invitrogen, CA, USA) containing 10% fetal calf serum (FCS, HyClone, UT, USA). Primary duck embryo fibroblasts (DEF) or primary chick embryo fibroblast (CEF) cells were prepared from embryonated unvaccinated duck eggs or SPF chicken eggs and cultured in M199 (Invitrogen, CA, USA) containing 4% FCS.

### Virus Mutagenesis

Based on the sequences of the NA and NS genes of Gs/GD/96, which possessed intact NA and NS genes, a 60-nucleotide fragment (TGC AAT CAA AGC ATT ATT ACT TAT GAA AAC AAC ACC TGG GTA AAT CAA ACA TAT GTC AAC, which is conserved in all H5N1 isolates) and a 15-nucleotide fragment (GCC ATT GCT TCC AGT, which is varied in different species-based isolates, the inserted 15 nucleotides can be found in chicken-, duck-, and goose- origin H5N1 viruses at 3.7%, 7.7%, and 62.5%, respectively) were inserted into the NA stalk and the NS1 genes of the AIV SY strain, respectively, through overlap PCR [30], [32], [33]. The primers used for the mutations are listed in Table 1. The modified NA and NS genes were cloned into the PHW2000 vector, verified through sequence analysis, and named pHW256-NA+ and pHW258-NS+, respectively. Virus rescue was performed as described previously [34], [35]. Briefly, eight rescue plasmids (pHW251-PB2, pHW252-PB1, pHW253-PA, pHW254-HA, pHW255-NP, pHW256-NA, pHW257-M, and pHW258-NS) [31] with or without the substitution plasmids pHW256-NA+ and/or pHW258-NS+ were cotransfected into a mixture of 293T and MDCK cells. After 48 h, the culture mixtures were inoculated into 10-day-old SPF eggs to amplify the rescued viruses at 35°C. The allantoic fluids were tested individually for the presence of infectious virus through a standard hemagglutination assay using chicken red blood cells (CRBCs) [36]. The RNAs of the propagated rescue viruses were extracted and amplified, and each viral gene segment was sequenced to ensure the absence of unwanted mutations. The rescue viruses were named A−S− if the virus exhibited both deletions in the NA and NS1 proteins, A+S− if the virus exhibited the 20-amino-acid insertion in the NA stalk, A−S+ if the virus exhibited the five-amino-acid insertion in the NS1 protein, and A+S+ if the virus exhibited both insertions in the NA and NS1 proteins.

**Table 1:** Primers for the mutagenesis of the NA and NS genes of the H5N1 AIV SY strain.

Primer name		Primer sequences (5′→3′)
mNA	Ba-NA-1^a^	TATTGGTCTCAGGGAGCAAAAGCAGGAGT
	NA-1d	TATGTCTGATTTACCCAGGTGTTGTTTTCATAAGTAATAATGCTT
		TGATTGCATGGTTCAACTTGGTGTTGATTCCCTGTCTGAATT
	NA-2u	ACTTATGAAAACAACACCTGGGTAAATCAGACATATGTC
		AACATCAGCAATACTAATTTTCTTACTGAGAAAGCTGTGGCTT
	Ba-NA-2	ATATGGTCTCGTATTAGTAGAAACAAGGAGTTTTTT
mNS	Bm-NS-1^b^	TATTCGTCTCAGGGAGCAAAAGCAGGGTG
	Bm-NS-1d	TTACGTCTCAATTGCCATTTTAAGTGCCTC
	Bm-NS-2u	TTACGTCTCGCAATTGCATCCAGCCCGACTTCAC
	Bm-NS-2	ATATCGTCTCGTATTAGTAGAAACAAGGGTGTTTT

Ba-NA-1^a^, the restriction endonucleases site for BsaI is underlined.

Bm-NS-1^b^ **,** the restriction endonucleases site for BsmBI is underlined.

### Growth Curve

Confluent MDCK, Vero, CEF, and DEF cells in 35-mm dishes were infected in duplicate with each rescue virus at a multiplicity of infection (MOI) of 0.01 and incubated at 37°C in the appropriate medium containing 1% FCS. The virus titers of the supernatants, which were collected at different time points, were determined as the number of 50% tissue culture infectious doses (TCID_50_) per 1 ml of CEF cell culture using the method described by Reed and Muench [37].

### NA Activity Assays

For the enzymatic assays, virus dilutions in U-bottomed microtiter plates were incubated with increasing concentrations (5 to 100 µM) of the fluorogenic substrate 4-methylumbelliferyl N-acetylneuraminic acid (4-MUNANA; Sigma, MO, USA), and the fluorescence of the released 4-methylumbelliferone was monitored using a Safire2 microplate reader (Tecan, Mannedorg, Switzerland). The kinetic parameters K_m_ and V_max_ were calculated by fitting the data to the appropriate Michaelis–Menten equations using KaleidaGraph software (Synergy Software) [11], [15], [33].

To determine the rate of virus elution from CRBCs, 50 µl of serial twofold dilutions of the viral stocks in phosphate-buffered saline (PBS) was incubated with 50 µl of 1% CRBC suspension in U-bottom microtiter plates. The plates were left on ice for 1 h to allow virus adsorption to the CRBCs and then transferred to a water bath at 37°C. The decrease in HA titer, which reflects the NA-mediated virus elution from CRBCs, was monitored for 24 h [15], [33].

### Antiviral Activity Assay of IFN-β

The antiviral activity of IFN-β was assayed as previously described [1], [38]. Briefly, Vero cells plated at a density of 2×10^5^ cells per well in 6-well plates were treated with recombinant human IFN-β (R&D systems, MN, USA) at different concentrations (100 U, 200 U, 400 U, 800 U, 1600 U, 2000 U, and 10,000 U) for 24 h in serum-free DMEM, and the cells were then inoculated with the viruses at an MOI of 0.0001. The culture supernatants were collected 72 h after inoculation for subsequent determination of the TCID_50_ per 0.1 ml of the CEF cell culture.

### Competition Inhibition Assay *in vitro*

Vero, MDCK, DEF, and CEF cells at a density of 2×10^5^ cells per well in 6-well plates were used for the serial passaging. The A−S− virus, which was mixed equivalently with the A−S+, A+S−, or A+S+ viruses (1×10^3^ TCID50 per 0.1 ml of each), was inoculated into the monolayer cells at an MOI of 0.01. After adsorption for 1 h at 37°C, the inoculum was removed, and fresh medium containing 1% FCS was added to the wells. The inoculated cells were incubated at 37°C for 24 h or 48 h according to the viral growth rate in the different cells. When approximately 80% of the cytopathic effect was obtained, the medium was collected and centrifuged at 800×g for 5 min at 4°C to remove the debris, and the supernatant was named the P1 stock. Each virus mixture stock was diluted 1000 (Hemagglutination titers ≤5log_2_) or 10,000-fold (Hemagglutination titer ≥ 6log_2_) with medium containing 1% FCS and passaged continually with the same cells up to the 10th passage. All of the supernatants were collected and stored at −70°C until use.

The total RNAs of the P1, P5, and P10 mixture samples from the different cells were prepared through treatment with the Trizol reagent (Invitrogen, CA, USA), and the full-length cDNAs of the viruses were synthesized using a 12-bp random primer [39]. The total viral RNA copies were quantified by quantitative real-time PCR (qRT-PCR) using the primers for the matrix gene, and the viral RNA copies of the A+S−, A−S+, and A+S+ viruses were quantified using the primers for NA and/or NS genes (one of the primer pairs was located in the insertion regions) (Table 2). The percentages of the A+S−, A−S+, or A+S+ viruses in the mixture of viruses were counted by comparing the copies of the single-mutant virus with that of the total viruses. All of the real-time PCR reactions were performed under the following conditions: 95°C for 30 s and 40 consecutive cycles of 95°C for 5 s and 60°C for 30 s. For all reactions, melting curve analysis was performed to verify the product specificity.

**Table 2:** SYBR green real-time PCR primers for the identification and quantification of the NA and NS genes containing or not containing amino-acid deletions in the viral cDNAs.

Target genes	Primer name	Primer sequences	Target sequencenumber
M gene	M-F	AAGTGGCTTTTGGCCTAGTGTG	EU195395 (422–443)
	M-R	TGATTAGTGGGTTGGTGATGGTT	EU195395 (498–520)
NA gene	NA-F^a^	GAAAACAACACCTGGGTAAATCAG	EU195394 (−)
	NA-R	CCATCCTCTAATGGGGCAAA	EU195394 (212–231)
NS gene	NS-F^b^	AATGGCAATTGCATCCAGC	EU195396 (−)
	NS-R	AACCTGCCACTTTCTGCTTGG	EU195396 (305–325)

NA-F^a^, the primer was targeted to the nucleotide sequence of the 20-amino-acid insertion in the NA stalk.

NS-F^b^, the primer was targeted to the nucleotide sequence of the five-amino-acid insertion in the NS1 protein.

### Expression Levels of Immune-related Genes in Peripheral Blood Mononuclear Cells of Mallard Ducks

The whole blood was collected from six-week-old ducks, and the peripheral blood mononuclear cells (PBMCs) were purified by treatment with lymphocyte separation media (Mediatech Inc., Herndon, VA, USA). The PBMCs in RPMI-1640 (Invitrogen, CA, USA) with 2% FCS were plated in six-well plates at 2×10^6^ cells per well and infected with each virus at an MOI of 1. The culture plates were gently rocked every 15 min for 1 h, and the media was then replaced with fresh media. The cells were harvested at 8 h postinfection, and the total RNA of these samples was extracted.

The quantification of the cytokine mRNA levels was performed according to the protocol described by Kuo et al. (2010) [40]. The primers for the IFN-α, MX1, IL-1β, IL-8, IL-10, IL-18, MHC-I, MHC-II, and TLR-7 genes of ducks were designed based on published sequences or previously reported primers [41], [42]. All of the primers are listed in Table 3. The expression level of each gene relative to that of GAPDH was calculated using the threshold cycle 2^–△△CT^ method [43].

**Table 3:** Real-time PCR primers for detection of the expression levels of immune-related genes in the PBMCs of mallard ducks.

Target genes	Forward primers	Reverse primers
GAPDH	ATGTTCGTGATGGGTGTGAA	CTGTCTTCGTGTGTGGCTGT
DMX1	TCACACGAAGGCCTATTTTACTGG	GTCGCCGAAGTCATGAAGGA
DIL-10	GGGGAGAGGAAACTGAGAGATG	TCACTGGAGGGTAAAATGCAGA
DIL-1β	GAGATTTTCGAACCCGTCACC	AGGACTGGGAGCGGGTGTA
DIL-8	AGGACAACAGAGAGGTGTGCTTG	GCCTTTACGATCCGCTGTACC
DIL-18	AGGTGAAATCTGGCAGTGGAAT	ACCTGGACGCTGAATGCAA
DIFN-α	TTGCTCCTTCCCGGACA	GCTGAGGGTGTCGAAGAGGT
DTLR-7	GTGGCAGCTTCAAGACAACA	TTAGTTGGCCATTCCAGGAC
DMHC- I	GAAGGAAGAGACTTCATTGCCTTGG	CTCTCCTCTCCAGTACGTCCTTCC
DMHC- II	CCACCTTTACCAGCTTCGAG	CCGTTCTTCATCCAGGTGAT

To determine the replication of the viruses in the duck PBMCs, the duck PBMCs were infected with each virus at an MOI of 1. The supernatant and cells were harvested at 4 h, 8 h, and 24 h. The numbers of the viruses were determined by quantifying the M gene copy numbers according to the above methods.

### Virulence in Chickens and Mallard Ducks

To determine the effect of A− and S− on the virulence of the rescue viruses, ten six-week-old SPF chickens were inoculated intravenously with 0.1 ml of a 1∶10 dilution of allantoic fluid and observed clinically over a period of 10 days. The intravenous pathogenicity index (IVPI) was determined according to the OIE standard [44]. The IVPIs of these viruses in six-week-old mallard ducks without AIV antibody were similarly determined.

To further determine the virulence of the viruses in ducks, six-week-old mallard ducks were randomly divided into six groups with 12 ducks per group. The ducks in groups 1 through 5 were inoculated intranasally with 0.1 ml of SY or one of the four rescue viruses at a dose of 1×10^6^ EID_50_, and the ducks in group 6 were challenged with sterile PBS as a negative control. On days 1, 3, 5, and 7 post-challenge, three ducks from each group were euthanized, and their heart, liver, spleen, lungs, kidneys, and brain were collected. The tissues samples were homogenized in PBS with antibiotics and titrated through inoculation in 10-day-embryonated chicken eggs. Oropharyngeal and cloacal swabs were collected from each group on days 3, 5, and 7 post-challenge. The swabs were placed immediately in PBS, and an aliquot was titrated through inoculation in 10-day-embryonated chicken eggs for the examination of virus shedding. All of the animals were housed in animal biosafety level 3 facilities at Yangzhou University.

### Statistical Analysis

The viral titers and viral loads are expressed as the mean ± standard deviation (SD). The expression levels of the immune-related genes are presented as the mean fold change ± SD. The statistical analyses were performed using an independent-sample *t* test. Differences with a *P* value of less than 0.05 were regarded as statistically significant.

## Results

### Prevalence of H5N1 Viruses with Double Deletions in NA and NS1 Proteins

All available sequences of the NA and NS1 genes from H5N1 viruses isolated between 1996 and 2012 were downloaded from GenBank, and the frequency of H5N1 viruses with double deletions in the NA and NS1 proteins was calculated. The results of the statistical analysis revealed that double deletions in the NA and NS1 proteins of H5N1 viruses were first found in 2002, and the numbers of these viruses were markedly increased in 2003. The ratio of H5N1viruses with double deletions in the NA and NS1 proteins was increased up to 90% in 2004 and thereafter (Table 4), which indicates that this type of virus has become predominant worldwide. In addition, the ratio of H5N1 viruses with double deletions in the NA and NS1 proteins isolated from land-based poultry was higher than that from domestic waterfowl in the early stage.

**Table 4:** Frequency of H5N1 viruses with double deletions in the NA and NS proteins from 1996 to 2012^a.^

Year	Ratios^b^						
	Viruseswith A−	Viruseswith S−	Viruses withA+ and S+	Viruses with A− and S−			
				Total	Land-basedpoultry	Domesticwaterfowl	Other sources^c^
1996–1999	10/16	0/16	6/16	0/16 (0^d^)	0/7 (0)	0/4 (0)	0/5 (0)
2000	3/21	5/21	13/21	0/21 (0)	0/0 (0)	0/11 (0)	0/10 (0)
2001	5/22	15/22	2/22	0/22 (0)	0/7 (0)	0/14 (0)	0/1 (0)
2002	20/29	21/29	1/29	13/29 (44.8%)	5/8 (62.5%)	4/12 (33.3%)	4/9 (44.4%)
2003	38/46	42/46	1/46	35/46 (76.1%)	13/15 (86.7%)	11/14 (78.6%)	11/17 (64.7%)
2004	104/110	102/110	3/110	99/110 (90%)	48/49 (98.0%)	19/23 (82.6%)	32/38 (84.2%)
2005	114/121	115/121	6/121	114/121 (94.2%)	46/46 (100%)	31/36 (86.1%)	37/39 (94.9%)
2006	147/153	148/153	5/153	147/153 (96.1%)	49/49 (100%)	25/27 (92.6%)	73/77 (94.8%)
2007	134/138	133/138	4/138	133/138 (96.4%)	53/54 (98.1%)	39/41 (95.1%)	41/43 (95.3%)
2008	75/76	73/76	1/76	73/76 (96.1%)	42/42 (100%)	12/13 (92.3%)	19/21 (90.5%)
2009	52/55	51/55	3/55	51/55 (92.7%)	30/30 (100%)	2/4 (50.0%)	19/21 (90.5%)
2010	48/48	43/48	0/48	43/48 (89.6%)	20/22 (90.9%)	10/11 (90.9%)	13/15 (86.7%)
2011	68/69	68/69	1/69	68/69 (98.6%)	15/15 (100%)	13/14 (92.9%)	40/40 (100%)
2012	10/10	10/10	0/10	10/10 (100%)	0/0 (0)	9/9 (100%)	1/1 (100%)

a All available sequences of both NA and NS1genes from H5N1 viruses deposited in Genbank were selected.

b The numbers indicate the ratio of the viruses to the total H5N1 viruses or the sources-based isolates in the indicated year.

c Other sources: The viruses from wild birds, mammals (including humans), and environmental samples.

d Percentage of H5N1 viruses with A− and S− isolated in the indicated year.

### Virus Rescue and Viral Replication in Different Cells

**Figure 1:**
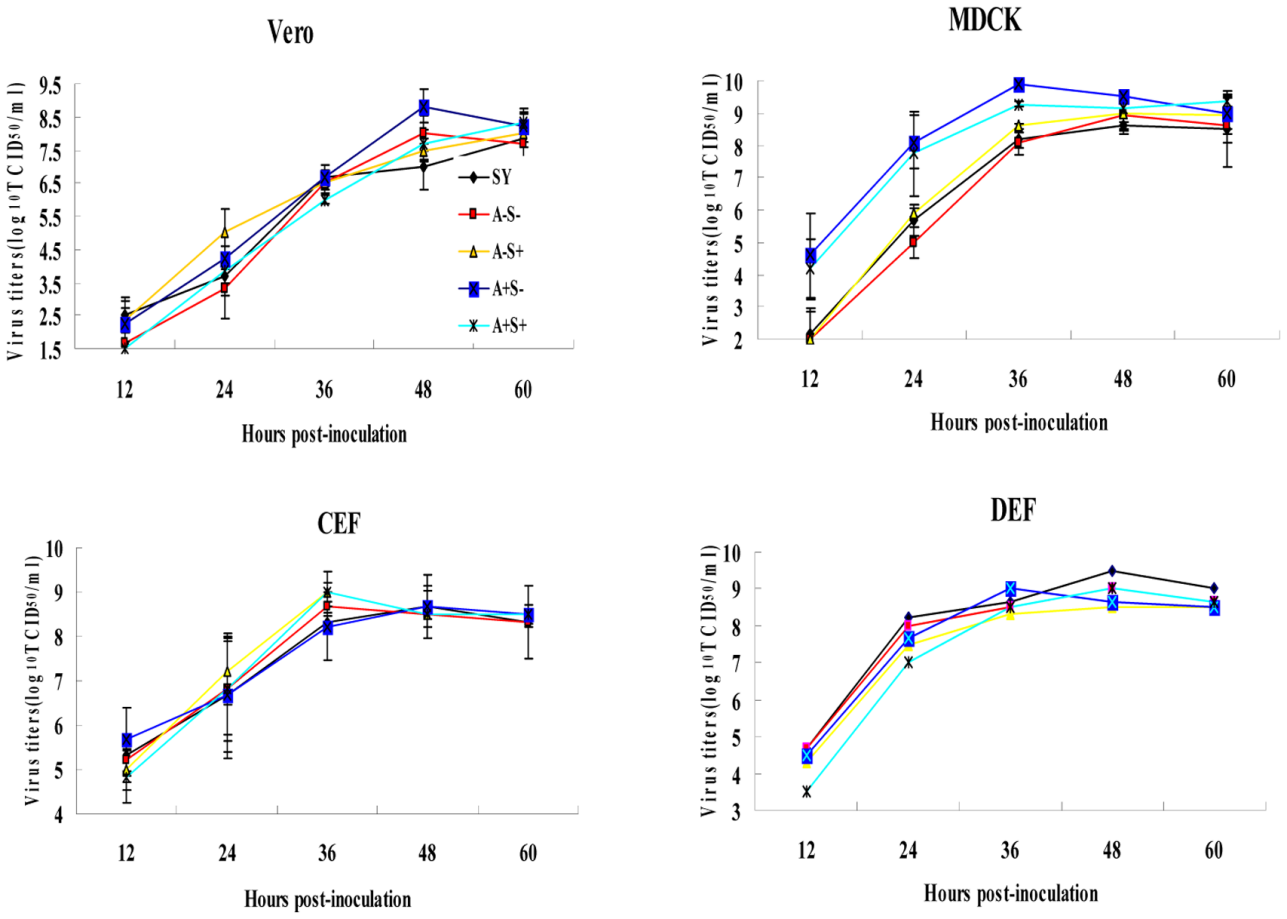
Growth kinetics of the viruses in Vero, MDCK, CEF, and DEF cells. The cells were infected with the wild-type strain and the four rescue viruses at an MOI of 0.01 TCID_50_/cell, and the culture media were harvested at the indicated times after infection. The virus titers at each time point are presented as the mean ± SD of duplicate experiments.

Four rescue viruses were generated. All of these viruses shared the same PB2, PB1, PA, HA, NP, and M genes derived from SY and carried different modified NA and/or NS genes. The TCID_50_ assay using CEF cells was performed to determine the replication kinetics of the viruses in Vero, MDCK, CEF, and DEF cells. As shown in Figure 1, the titers of the four viruses were similar to each other in Vero or CEF cells. However, the titers of A+S− and A+S+ in MDCK cells were approximately 1.5 log_10_ TCID_50_/ml higher than those of A−S− and A−S+, and the titers of A+S+ in DEF cells were approximately 0.5 log_10_ TCID_50_/ml lower than those of the other three viruses at 12 and 24 h postinfection. The wild-type strain SY displayed a similar growth pattern as the rescue A−S− in the four types of cells. These results suggest that both A− and S− can improve the viral replication in DEF cells at the early stages of AIV infection.

### Enzymatic Activity of the Neuraminidase

To evaluate the possible effects of A− and S− on the neuraminidase activity, the enzymatic parameters of SY and the four rescue viruses were determined using the MUNANA fluorogenic substrate. As shown in Table 5, the K_m_ values, which reflect the affinity for the substrate, for the viruses with A− were very similar (*P*>0.05) but approximately 1.4- to 2.9-fold lower (reflecting a higher affinity) than those obtained for the viruses with long-stalk NA (*P*<0.05). In addition, the V_max_ values, which depend on both the specific activity and the amount of enzyme in the reaction, for the viruses with A− were 1.56- to 2.01-fold lower than those obtained for the viruses with long-stalk NA (*P*<0.05), which indicates that A− decreases the enzymatic activity of the neuraminidase toward small MUNANA substrates.

**Table 5:** Enzymatic properties of the NA protein of H5N1 viruses.

Virus	K_m_ (µM)^a^	V_max_ (fluorescence U/S)^a^	V_max_ ratio^b^	Elution time (h)
SY	275.57±8.62	13.02.33±0.27	1.00	12
A−S−	192.7±3.35	15.69±0.56	1.20	12
A−S+	244.9±3.37	10.49±0.09	0.81	12
A+S−	551.2±19.8	26.18±0.58	2.01	6
A+S+	381.97±2.9	20.36±0.12	1.56	6

a The results are presented as the mean ± SD from three independent determinations on duplicate samples using dilutions of the H5N1 viruses.

b V_max_ ratio of the rescue viruses to the wild-type SY virus.

The elutions of the viruses from CRBCs were also determined. The complete elution of the viruses with long-stalk NA occurred within 6 h, whereas the viruses with short-stalk NA were completely eluted from the CRBCs after a 12-h incubation at 37°C. This finding indicates that A− reduced the rate of viral elution from CRBCs.

### IFN Resistance of the Rescue Viruses

To evaluate the resistance of the viruses to IFN, Vero cells were pretreated with different concentrations of IFN and infected with the viruses. The replication of A+S+ in Vero cells was completely inhibited in the presence of IFN-β at a concentration of 400 U, and the replication of A+S− was fully inhibited at an IFN-β concentration of 1600 U. However, the titers of A−S+ and A−S− were still detectable in the presence of IFN-β at a concentration of 10,000 U (Table 6), which indicates that A− and S− both enhance the interferon resistance of the viruses and that A− plays a more important role.

**Table 6:** IFN-β resistance of H5N1 viruses.

Viruses	Titers after pretreatment with different concentrations of IFN-β^a^							No treatment
	^100 U^	^200 U^	^400 U^	^800 U^	^1600 U^	^2000 U^	^10,000 U^	
SY	7.67	7.5	6.67	6.67	4.5	4.5	2.67	7.5
A−S−	7.33	7	7	6.33	4.67	3.67	1.33	7
A−S+	7	6.5	6.5	6	4.33	4.33	1.83	7.5
A+S−	6.67	7	6.67	5.33	<	<	<	6.5
A+S+	6.5	6.33	<	<	<	<	<	6.67

a Vero cells were pretreated with different concentrations of recombinant human IFN-β at 37°C. After 24 h, the cells were infected with the viruses at an MOI of 0.0001. The virus titers (log_10_TCID_50_/0.1 ml) were measured 72 h after infection. The values indicate the means of three experiments. <: titer <0.5.

### SYBR Green Real-time PCR Assay

A mixture of cDNAs of the A−S− and A+S+ viruses at the same concentration of approximately 4.00×10^4^ copies/µl was used to test the specificity and accuracy of the SYBR green real-time PCR assay. The results indicated that the average amount of the NA gene without deletion (from the A+S+ virus) was 1.98×10^4^ copies/µl. The average amount of the NS gene without deletion (from the A+S+ virus) was 1.97×10^4^ copies/µl, and the average amount of the M gene (from the A−S− and A+S+ viruses) was 3.97×10^4^ copies/µl. The percentage of the A+S+ virus was approximately 49.75%, and the percentage of the A−S− virus was approximately 50.25% (*P*>0.05). In addition, the plasmids pHW256-NA+, pHW258-NS+, and pHW257-M at the concentrations of 4.5×10^6^ copies/µl, 6.0×10^5^ copies/µl, and 3.3×10^6^ copies/µl, respectively, were evaluated by assays for five replicate tests, and the average concentrations were 4.504×10^6^ copies/µl, 5.982×10^5^ copies/µl, and 3.302×10^6^ copies/µl for each plasmid, respectively. Furthermore, the plasmids mixtures (pHW256-NA+ and pHW256-NA, or pHW258-NS+ and pHW258-NS) were tested using the assays, and only the copy numbers of plasmids pHW256-NA+ or pHW258-NS+ were detected (data not shown). These data indicate that the SYBR green real-time PCR method can efficiently detect the proportion of the viruses with intact NA or NS genes in the virus mixtures of interest.

### Competitive Growth on Different Cells

**Figure 2:**
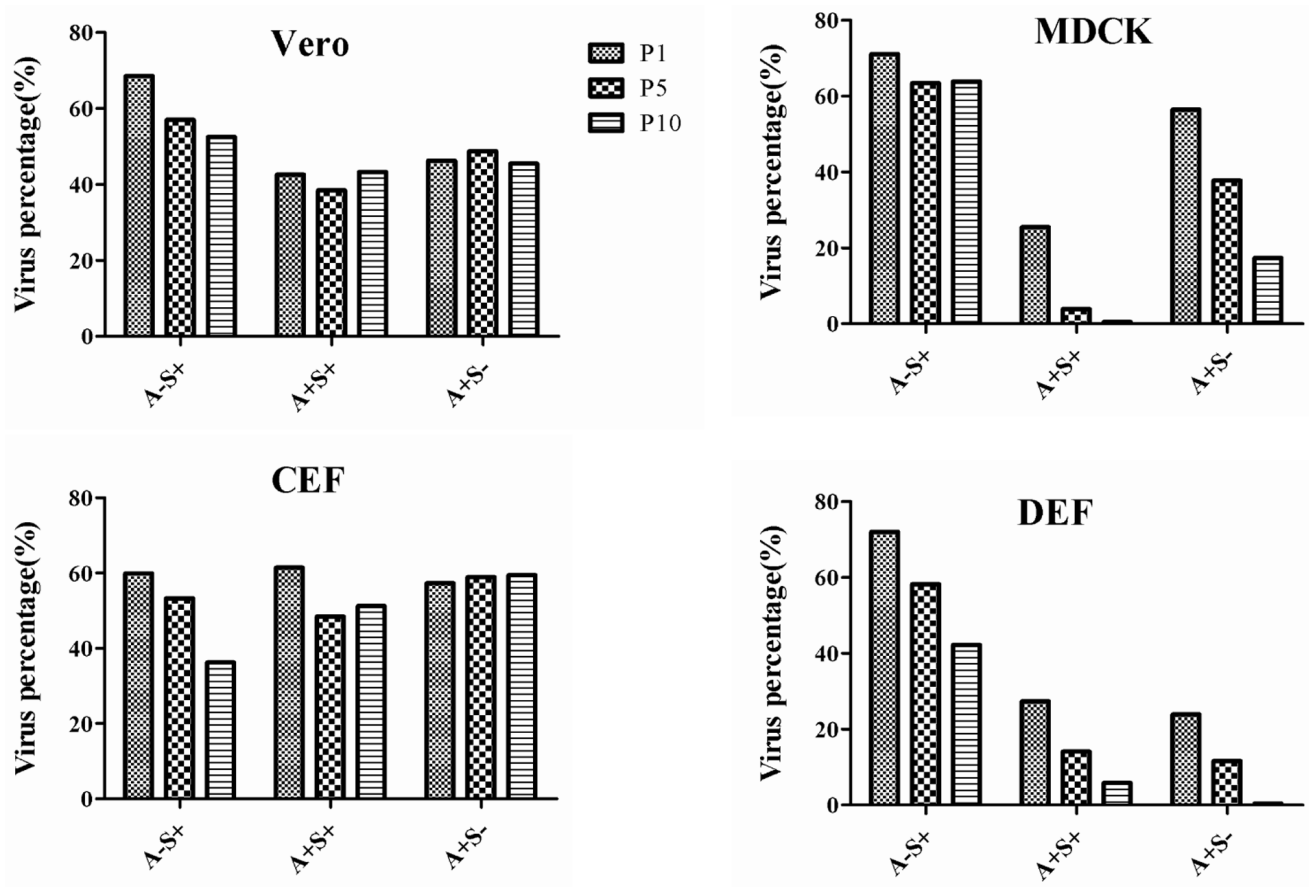
Serial passage of the mixture of two rescue viruses in Vero, MDCK, CEF, and DEF cells. The A−S− virus was mixed with A−S+, A+S−, or A+S+ (approximately 1×10^3^ TCID50 per 0.1 ml of each virus), and the mixture was inoculated into different cells and then serially passaged for 10 generations. The percentages of A−S+, A+S−, and A+S+ in the P1, P5, and P10 samples of the different cells were detected by SYBR green real-time PCR assay.

The A−S− virus, which was mixed with A−S+, A+S−, or A+S+, was serially passaged in Vero, MDCK, CEF, and DEF cells for ten generations. The cDNAs of the P1, P5, and P10 samples from different cells were detected using the above-described SYBR green real-time PCR assay. The results indicated that the viral percentage of A−S− in the P1, P5, and P10 mixture of A−S− and either A+S+ or A+S− were not significantly different from each other in Vero and CEF cells, whereas the percentages of A+S+ and A+S− in the P10 samples obtained from the MDCK cells were 1.5% and 17.4%, respectively, and the percentages of A+S+ and A+S− in the P10 samples from the DEF cells were 5.8% and 0.5%, respectively (Fig. 2). The percentage of A−S− in the mixture of A−S− and A−S+ was increased slightly after serial passage in DEF and CEF cells. These data indicate that the A−S− virus replicates predominantly in DEF cells that are co-infected with other variants.

### Expression Levels of Immune-related Genes in the PBMCs of Mallard Ducks *in vitro*

**Figure 3:**
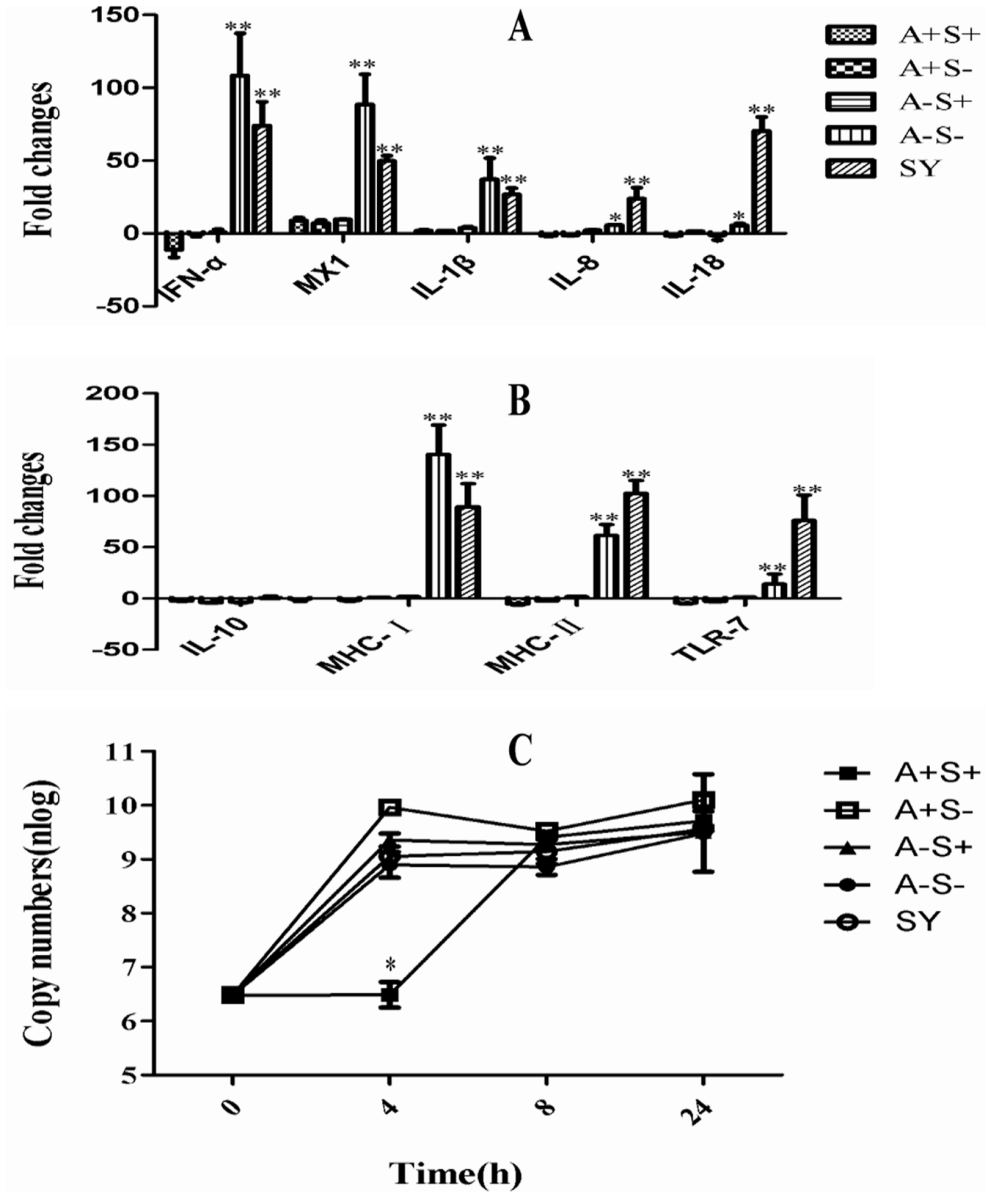
Real-time RT-PCR quantification of the expression of immune-related genes in mallard PBMCs and growth kinetics of the viruses in mallard PBMCs. Mallard PBMCs in a six-well plate were inoculated with SY and the four rescue viruses at an MOI of 1 TCID_50_/cell. The total RNA was extracted from the PBMCs at 8 h postinfection, and equal amounts of RNA (1 µg) from each sample were used for RT-PCR. The gene expression was normalized to the expression level of the GAPDH gene and is presented as the fold increase relative to the results observed with mock-treated cells. The data represent the mean fold changes ± SD (A and B). Mallard PBMCs were also infected with these viruses at an MOI of 1 copy/cell, and the supernatant and cells were harvested at 4 h, 8 h, and 24 h post-infection. These samples were determined by quantitative real-time PCR (qRT-PCR) using the primers for the matrix gene. The numbers of the viruses are presented as the mean ± SD of duplicate experiments (C).

To investigate the effect of the four rescue viruses on the host response, the PBMCs of mallard ducks were challenged with the viruses, and the induced expression levels of immune-related genes were determined at 8 h postinfection. There was no significant difference in the expression level of the anti-inflammatory cytokine IL-10 among the PBMCs infected with the different viruses. In contrast, the expression levels of the IFN-α, MX1, IL-1β, IL-8, IL-18, MHC-I, MHC-II, and TLR-7 genes in the PBMCs infected with SY and A−S− were significantly increased. There was slight upregulation or downregulation of the expression of immune-related genes in the PBMCs infected with the other variants (Fig. 3A, 3B). These results indicate that the virus with both A− and S− induced higher expression of the immune-related genes in PBMCs. When the growth curves of the viruses were determined in the PBMCs, only A+S+ displayed a significant delay in growth rate at 4 h postinfection, and there were no significant difference in growth rate among the other three viruses (Fig. 3C), which displayed similar trends in DEF cells.

### Virulence in Chickens and Mallard Ducks

To determine the effect of A− and S− on viral virulence, chickens or mallard ducks were challenged intravenously with the four rescue viruses. The IVPIs of the viruses in chickens ranged from 2.96 to 3.00, which indicates that all of these viruses are highly pathogenic to chickens. However, the IVPIs of A+S+, A+S−, A−S+, and A−S− in ducks were 0.054, 1.336, 1.307, and 2.314, respectively, which indicates that A+S+ is slightly pathogenic to mallard ducks, whereas A−S+, A+S−, and A−S− are all highly pathogenic to mallard ducks (the A−S− virus was the most virulent strain to mallard ducks).

**Figure 4:**
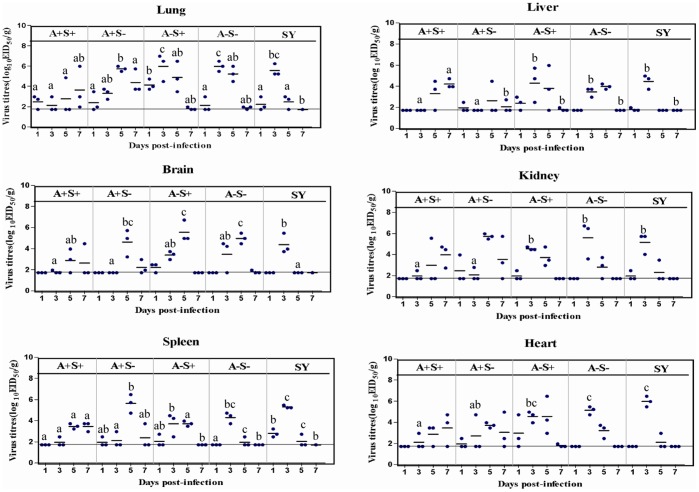
Figure 4. Replication kinetics of SY and the four rescue viruses in mallard ducks. The virus titers in the lungs, livers, hearts, spleens, kidneys, and brains of mallard ducks inoculated intranasally with 10^6^EID_50_/0.1 ml of SY and the four rescue viruses were determined. Each horizontal bar represents the mean virus titer in log_10_ EID_50_/g of tissue. The horizontal line indicates the lower limit of detection. Different lowercase letters indicate significant differences among SY and the four viruses infected groups on the same day postinfection (*P*<0.05). For example, on day 3 postinfection, the mean viral titers in the lungs of A+S+ (a) and A+S− (ab) groups were significantly lower than that of A−S+ (c) and A−S− (c) groups, and there were no significant differences between the A+S− (ab) and SY (bc) groups. There were also no significant differences among the A−S− (c), A−S− (c) and SY (bc) groups.

Because the virulence of the four viruses exhibited differences in mallard ducks through the intravenous route, mallard ducks were challenged intranasally with the four viruses, and the viral loads in the main organs of the infected ducks were determined by viral culture. The mean viral titers in the lungs, livers, kidneys, and spleens of A+S+ or A+S–infected ducks was lower than the mean viral titers detected in the groups infected with A−S+, A−S−, or SY on day 3 postinfection (*P*<0.05). The mean viral titer in the hearts of the A+S+-infected mallard ducks was lower than that obtained in the hearts of the ducks infected with A−S+, A−S−, or SY on day 3 postinfection (*P*<0.05). The mean viral titers in the brains of A+S+-infected ducks was lower than the mean viral titers detected in the groups infected with A−S+, or A−S− on day 5 postinfection (*P*<0.05) (Fig. 4). In addition, in both A+S+ and A+S− groups, virus replication appears to be delayed in most organs, compared with that measured in both the A−S+ and A−S− groups.

Viral shedding was detected in both oropharyngeal and cloacal swabs from infected ducks on days 3, 5, and 7 postinfection. On day 3 postinfection, the viral shedding ratios obtained from the oropharyngeal swabs from the groups infected with A+S+, A+S−, A−S+, and A−S− were 22.2% (2/9), 44.4% (4/9), 100% (9/9), and 88.9% (8/9), respectively (Table 7), which was correlated with the viral titers in the lungs at the same time point (Fig. 4). On day 7 postinfection, the viral shedding ratios obtained from the oropharyngeal swabs from group infected with A+S+ and A+S− were 100% and for both, which was also correlated with the higher viral titers in lungs at same time point. However, at this time point (on day 7 postinfection), there was no detectable viral shedding in both the A−S+− and the A−S− infected groups. Compared with the ducks infected with A+S+ and A+S−, the viral shedding of ducks infected with A−S+ and A−S− reached a peak two days earlier. On day 3 postinfection, the viral shedding ratios in the cloacal swabs samples from ducks infected with A+S−, A−S+, or A−S− were 11.1% (1/9), 66.7% (6/9), and 33.7% (3/9), respectively. However, there was no detectable viral shedding in the cloacal swabs samples from ducks infected with A+S+. These results indicate that mallard ducks infected with A+S+ shed the progeny virus only through the oropharynx, whereas mallard ducks infected with A+S−, A−S+, and A−S− shed the progeny virus through both the oropharynx and cloaca.

**Table 7:** Virus shedding of oropharyngeal and cloacal swabs of mallard ducks inoculated with 0.1^6^ EID_50_ H5N1 viruses.

Virus	NO. of positive swabs/NO. of total swabs (log_10_EID_50_± SD)					
	Oropharyngeal swabs			Cloacal swabs		
	3 dpi^A^	5 dpi	7 dpi	3 dpi	5 dpi	7 dpi
A+S+	2/9(3.00±0.71)	4/6(3.63±0.97)	3/3(3.00±0.50)	0/9(<0.5)	0/6(<0.5)	0/3(<0.5)–
A+S−	4/9(2.38±0.92)	6/6(2.79±0.86)	3/3(1.58±1.23)	1/9(1.5)	2/6(1.75±0.01)	2/3(1.63±1.24)
A−S+	9/9(3.22±1.37)	6/6(2.50±1.22)	0/3(<0.5)	6/9(1.33±0.79)	3/6(1.50±0.66)	0/3(<0.5)
A−S−	8/9(3.16±0.88)	4/6(2.13±0.92)	0/3(<0.5)	3/9(1.67±0.14)	0/6(<0.5)	0/3(<0.5)
SY	8/9(2.81±0.40)	4/6(2.81±1.20)	0/1 B(<0.5)	7/9(1.32±0.45)	1/6(1.25)	0/1 (<0.5)

The oropharyngeal and cloacal swabs were collected for virus isolation from each group at 3, 5, and 7 days postinfection.

A dpi: days postinfection.

B Two out of three ducks were dead on day 6 postinfection.

## Discussion

According to the deletion length and location in the NA stalk, H5N1 viruses isolated in 1997 were divided into four groups: a long NA stalk, a short NA stalk with a 20-amino-acid deletion at positions 49 to 68, a short NA stalk with a 20-amino-acid deletion at positions 55 to 74, and a short NA stalk with a 19-amino-acid deletion at positions 55 to 73. Since that time, only long NA stalks and short NA stalks with a 20-amino-acid deletion at positions 49 to 68 have been observed in H5N1 viruses, which indicates that the other two types of viruses had a selective evolutionary disadvantage. H5N1 viruses with a short NA stalk and a five-amino-acid deletion from position 80 to 84 in the NS1 protein were first observed in 2002, became predominant in 2003, and have continued to exhibit a very high ratio (approximately 90%) in subsequent isolates. In addition, the deletion in both NA and NS1 proteins of H5N1 viruses was biased for land-based poultry in the early stage. However, there were few isolates of other subtypes of influenza virus that have contained the double deletions in the NA and NS1 proteins. It is possible that the H5N1 viruses with double deletions in the NA and NS1 proteins have a prevailing advantage and are stably maintained in poultry. It is worthwhile to note that H5N1 viruses have been found to be highly pathogenic to ducks since 2002 [45], [46]. To investigate the role of double deletions in the NA and NS1 proteins in the pathogenicity of H5N1-subtype AIVs, a series of rescue viruses, which were derived from a H5N1 AIV with double deletions in the NA and NS1 proteins, was obtained by reverse genetics. We found that these rescue viruses all replicated efficiently in embryonated chicken eggs, which indicates that the presence or absence of the deletion in the NA stalk and the NS1 protein of H5N1 viruses did not significantly change their viral replication ability in embryonated chicken eggs.

In accordance with previous reports [33], [47], at the early stage of viral infection, the titers of A+S− and A+S+ in MDCK cells were approximately 1.5 log_10_ TCID_50_/ml higher than those of A−S− and A−S+, which indicates that the replication ability of the viruses with a long-stalk NA in MDCK cells was better than that of the viruses with a short-stalk NA. The enzymatic activities of the neuraminidase of viruses with a long-stalk NA were higher than those of viruses with a short-stalk NA, as judged by their higher rates of elution from CRBCs. Thereafter, higher NA activity facilitated the release and diffusion of progeny virions, which resulted in a higher replication ability of the viruses with a long-stalk NA in MDCK cells. MDCK cells express high amounts of both α2,3 and α2,6-gal sialyl glycoconjugates [48], [49]. Vero cells express a high amount of α2,3-linked receptors and a relatively low amount of α2,6-gal-linked receptors, and avian cell lines (QT-6 and DF-1) express a high amount of α2,3-gal-linked receptors [48]. Thus, the match of the viral NA activity and the viral binding ability to cellular receptors contributes to the replication ability of H5N1 viruses in different cell lines. It is thus reasonable that the growth pattern of the viruses in Vero, CEF, and DEF cells are different from that in MDCK cells.

Vero cells are IFN-α/β-deficient, and the replication abilities of the four rescue viruses in Vero cells were similar. However, there was a significant difference in viral growth in the IFN-β-pretreated Vero cells, which indicates that the interferon-resistance abilities of these viruses were different. The order of the interferon resistance ability from high to low of these viruses was A−S+ = A−S−>A+S−>A+S+. These results suggest that both A− and S− enhance the interferon-resistance ability of H5N1 AIVs. It has been reported that the NS1 protein is critical for the influenza virus to antagonize the host cell IFN response [1], [23], [50]. However, there is no report on the NA protein of influenza virus participation in the viral resistance to the host IFN response. Therefore, the mechanism through which the short-stalk NA protein counteracts the anti-viral activity of IFN-β needs to be further investigated.

It has been reported that RIG-I expression is an intracellular RNA sensor that detects the presence of vRNA, leading to induced expression of IFN-β [51]. CEF cells are derived from chickens, which lack RIG-I [52], and may, therefore, fail to induce the expression of IFN-β via this pathway. Further study confirmed that no IFN expression was observed in CEF cells infected with avian influenza viruses [53]. This may explain that the viral percentage of A−S− in the mixture of A−S−, and A+S+ or A+S− were not significantly different from each other in CEF cells and IFN-deficient Vero cells in the competition assay. However, RIG-I expression is detected in both MDCK cells [54] and duck cells [52]. Although both A+S+ and A+S− displayed higher replication ability in MDCK cells when compared with A−S−, A−S− replicated dominantly when co-infected with A+S− or A+S+ in MDCK and DEF cells. The interferon-resistance ability of A−S− virus might contribute to its replication predominance to some extent, and the precise mechanism of the replication advantage of the A−S−virus over the A+S+ virus in the competition assay needs to be further studied.

To evaluate the effect of A− and S− on the viral pathogenicity in poultry, the IVPIs of these viruses in chickens and mallard ducks were measured. It has been reported that a deletion in the NA stalk of H1N1 AIV results in increased virulence for chickens [11], and a deletion in the NA stalk of H5N1 AIV results in no significant difference in the virulence for mice via the intranasal route [9]. Because of the high pathogenicity of the parental virus SY in SPF chickens, the IVPIs of the A−S− virus and its variants for chickens were all similar and thus did not reflect the effect of A− and S− on the viral pathogenicity of these viruses in chickens. However, the IVPIs of A−S+ and A+S− for mallard ducks were significantly higher than that of A+S+ and lower than that of A−S−, which indicates that both A− and S− result in a marked increase in the virulence of the viruses for mallard ducks. In addition, this finding demonstrated that A− and S− exert a synergistic effect on the virulence of H5N1 viruses for mallard ducks. We also found that the PBMCs of mallard ducks infected with A−S− displayed a significant cytokine response, although the growth rate of A−S− was similar to that of A−S+ or A+S−. It was hypothesized that the high expression levels of IFNs and proinflammatory cytokine genes in PBMCs may play an important role in the high pathogenicity of A−S− to mallard ducks through the intravenous route.

We also monitored the viral pathogenicity in mallard ducks after intranasal inoculation. Different from the intravenous inoculation, the viruses (except for SY with two deaths out of nine ducks) at dosages of 1×10^6^ EID_50_ caused serious clinical signs but no death in mallard ducks within the observation period. Compared to the A+S+-inoculated mallard ducks, the mallard ducks infected with A−S+, A+S−, and A−S− presented higher virus titers in the lungs and brain. Furthermore, compared with A+S+ or A+S−, A−S+ and A−S− displayed faster replication ability in the lungs of mallard ducks. In addition, the viral shedding results demonstrated that mallard ducks infected with A+S+ shed the progeny virus only through the larynx, whereas mallard ducks infected with the other viruses shed the progeny virus through not only the larynx but also the cloaca. It is worth determining the viral replication ability in the intestines of ducks in a future study. These data suggest that with stronger ability to resist the interferon inhibition (as shown in Table 6), the A−S− and A−S+ viruses possessed stronger ability to overcome or suppress the host immune system and achieved increased viral replication ability. In addition, both A− and S− enhanced the viral replication ability and shedding of H5N1-subtype AIVs in mallard ducks and the S− of H5N1 viruses had less of an effect on the virulence than A− when the virus infection occurred via the intranasal route.

In summary, H5N1 AIVs with double deletions in the NA and NS1 genes have been the prevailing strains in recent years. The rescue virus with both a short-stalk NA and a deletion in the NS1 protein exhibited increased interferon resistance, competitive inhibition in DEF cells, increased expression of immune-related genes in the PBMCs of mallard ducks, and increased virulence in mallard ducks compared with the rescue virus with intact NA and NS1 protein. Although H5N1 AIVs with double deletion in the NA and NS1 genes most likely occur in chicken in nature but not in waterfowl, the genotype is maintained in waterfowl once the virus is re-introduced [9], [30]. Our data indicate that both deletions in the NA stalk and the NS1 protein contribute to the high pathogenicity of H5N1 AIVs in ducks and that these deletions may play an important role in the maintenance and circulation of these viruses in poultry.
